# *Candida albicans* Beta-Glucan Induce Anti- Cancer Activity of Mesenchymal Stem Cells against Lung Cancer Cell Line: An *In-Vitro* Experimental Study

**DOI:** 10.31557/APJCP.2020.21.3.837

**Published:** 2020-03

**Authors:** Fatemeh Peymaeei, Fatemeh Sadeghi, Elahe Safari, Samaneh Khorrami, Mehraban Falahati, Shahla Roudbar Mohammadi, Maryam Roudbary

**Affiliations:** 1 *Department of Medical Mycology and Parasitology, *; 2 *Department of Immunology, School of Medicine, *; 3 *Immunology Research Center, Institute of Immunology and Infectious Diseases, Iran University of Medical Sciences, *; 4 *Department of Mycology, Faculty of Medical Sciences, Tarbiat Modares University, Tehran, Iran. *

**Keywords:** β-glucans, lung cancer, mesenchymal stem cells, apoptosis

## Abstract

**Objective:**

β-glucan, glucopyranosyl polymers of fungi cell wall, represent an immune stimulating effects with potential anti-cancer activity. Mesenchymal stem cells (MSC) have immunomodulating properties in cancer microenvironment. The aim of this study was to investigate the anti-cancer effect of *Candida albicans* *(C. albicans) *beta-glucan on MSCs supernatant for apoptosis assay of lung cancer cells in vitro.

**Methods:**

Beta-glucan was extracted from cell wall of C.albicans. MSC isolated from adipose tissue of patients and confirmed using specific surface markers expression which examined by flow cytometry. MSCs treated with various concentrations of β-glucans for 48 hours. Cytotoxic effect of β-glucans was evaluated using MTT assay. MSC and lung cancer line cocultured and treated with β-glucans and apoptosis assay was done by flow cytometry.

**Results:**

Cytotoxicity findings showed a significant decrease in MSC viability during 48h, however it was dose-dependent (P<0.05). According to the obtained findings, supernatant of mesenchymal stem cells treated with β-glucans increased cancer cells apoptosis (P<0.05).

**Conclusion:**

Beta glucan may highlight a potential and novel promising candidate in future strategies to cause apoptosis of cancer cells and consider as therapeutic agent against tumor growth as well. Definitely, more in vitro and in vivo studies are required to understand its functions.

## Introduction

β-Glucans are composed of repeating glucose monomer units linked by β-1,3-glycosidic bonds (Yamada et al.,2009). Moreover, fungi have a β-Glucan rich cell wall compromised of glucose residues arranged in β (1–3) D glucopyranosyl polymers and β (1–6) D glucopyra-nosyl side chains with varying length and frequency distribution (Lebron et al., 2003). However, beta glucans, which are isolated from different sources exhibited different structures and solubility, but interestingly, they have similar biological properties (Sawai et al., 2002). 

Several investigations in animals and humans have subsequently shown that β-glucans, either particulate or soluble, represent immune stimulating properties, including anti-bacterial and anti-tumor activities through different pathways like activation of immune cells to suppress cancer cell proliferation (Choromanska et al., 2018; Sima et al., 2019; Vetvicka et al., 1996). The existence of special membrane receptors on leukocytes for β-glucan mediate immunomodulating function. β-glucans primarily activate innate immune system such as macrophage, Dendritic cells and secretion pro-inflamatory cytokines, subsequently activation adaptive immune cells specifically various T cells subtypes in tumor micro environment, ultimately destruct tumor cells,leading to cell death. (Herre et al., 2004; Schorey et al., 2008; Taylor et al., 2002).

Mesenchymal stem cells (MSCs) are fibroblast-like cells and they can be easily isolated from bone marrow, adipose, and other tissues including cord blood, peripheral blood, fetal liver, skeletal muscle, placenta, amniotic fluid and synovium (Campagnoli et al., 2001, Scherjon et al., 2003; Zuk et al., 2002; Erices et al., 2001; De Bari et al., 2002; Kuznetsov et al., 2001;Tondreau et al., 2005). In other words, MSCs are a subset of non-hematopoietic adult stem cells that originate from the mesoderm. They possess self-renewal ability and multi-lineage differentiation into not only mesoderm lineages, such as chondrocytes, osteocytes and adipocytes, but also ectodermic cells and endodermic cells (Wei et al., 2013).

Recently, laboratory and clinical data on the use of MSCs have fueled an increasing interest in diverse fields, especially for cancer. So that, MSCs can also suppress tumor growth by cell cycle arrest and inhibition of proliferation. Anti-tumor properties are described for MSCs isolated from various sources in experiments both in vitro and in vivo of various tumors. To date, there are large number of experimental studies that confirm the anti-oncogenic potential of MSCs modified with therapeutic genes and/or loaded with chemotherapeutic drugs (Chulpanova et al., 2018). Nonetheless, it has been shown that MSCs can also home to tumor sites and contributes to tumor growth and progression. It is not obvious however, whether the MSC effect is predominantly tumor promoting or suppressive (Ridge et al., 2017). However, MSC have immune modulating properties that mostly suppress the immune response by production of inhibitory mediators such as IDO, TGF- β, PGE-2 and IL-10.

Lung cancer is the most common cancer in terms of both incidence and cancer-related mortality worldwide (Roudi et al., 2017). 

 Although significant progress has been made in the understanding of disease pathogenesis and development of novel therapies, lung cancer remains an incurable disease. Lung cancer is a heterogeneous disease categorized into two main types: small-cell lung cancer (SCLC) and non-small-cell lung cancer (NSCLC) (Liu et al.,2014). Until now few clinical trial for beta-glucans in lung cancer patient was carried out and continued (Weitberg et al., 2008; Gao et al., 2005). However, the anti-cancer activity of beta-glucans against cancer cells was shown in the previous published data but the efficacy of this component have not been reported on MSCs until now, to raise this issue, in the current study, we sought for the first time to assess the effect of beta glucan extracted from *C. albicans *cell wall on MSC cells for the induction of apoptosis, in vitro (Roudi et.al.,2017) and (Geller et.al.2019).

## Materials and Methods

*β (1,3) Glucan extraction of Candida albicans Cell wall and characterization*

β-glucan was extracted from *C. albicans *cell wall (ATCC-10231) as previously described by (Nasrollahi et al., 2013) with some modifications. Solubilized β-glucans were obtained by oxidation of the *Candida* cell wall with sodium hypochlorite and sodium hydroxide (SDA, Merck). The particles solubilized by treatment with dimethyl sulfoxide (DMSO) (sigma) and zymolyase (sigma) digestion to extract β (1,3) D-glucan (20 mg beta glucan suspended in 10 mL of acetate buffer (50 mM, pH 6.0) was mixed with 1 mg of Zymolyase 100T enzyme and also parentheses. The soluble fractions were lyophilized. Proton magnetic resonance (H NMR) was used to examine the extracted beta-glucan. The sample was dissolved in Deuterium oxide (D2O) (sigma) and NMR spectrum was recorded on a Bruker AVANCE 300 FT-NMR spectrometer, resonating at 300 MHz (1 H) and compared with the 1H NMR spectrum of standard form of beta glucan. 

*Cell Line*

A549 cell line (Lung Adenoma carcinoma cells) was purchased from National Center for Genetic and Biological Reserves of Iran. The cells cultured on high glucose Dulbecco’s modified Eagle’s medium (DMEM, Gibco, USA) containing 10% fetal bovine serum (FBS) (Gibco, USA) in a humidified 37°C incubator with 5% CO_2_.

*Isolation of mesenchymal stem cells*

Adipose tissue was isolated from patients undergoing surgery. The adipose tissue was washed three times with PBS (Gibco, USA) buffer composed of penicillin/streptomycin (Gibco, USA) and digest by collagenase type 4 for 30 hours at 37°C. After passing through 0.7 micron filter and removing the portion containing RBC, the cells were cultured in DMEM medium (Gibco, USA) containing 10% FBS (Gibco, USA), 1% Penicillin and Streptomycin and 200mM sterile-filtered L-Glutamine (Gibco, USA). MSCs passaged two and three times and were used for tests.

*Characterization of MSC*

Primary MSCs were isolated from human abdominal fat (5-10g) using collagenase-H, cultured for 3 weeks in advanced MEM medium (Gibco, USA) supplemented with platelet lysate (PLTmax, Mill Creek Life Sciences, Rochester, MN), and used at the third passage, as previously shown. Cells were characterized in vitro by flow cytometry analysis for CD45, CD11b, CD90, and CD105 using FACS Calibur (Becton, Dickinson and Company, Franklin Lakes, NJ) as previously described (Campagnoli et al., 2001; De Bari et al., 2002). 

*Osteogenic differentiation*

The MSCs were trypsinized, counted and reseeded at 5,000 cells/cm^2^ culture flask. The medium was replaced with osteo¬genic medium (low-glucose DMEM containing 5% FBS, 10 nM dexamethasone, 50 μM L-ascorbic acid-2-phosphate and 10 mM glycerophosphate; (all from Sigma-Aldrich) and cells incubated at 37^o^C in a humidified 5% CO_2 _atmosphere. After 21 days, mineralization was measured using Alizarin Red S (Sigma) staining. Briefly, the MSC cultures were fixed in a 4% paraformaldehyde solution and stained with a 0.5% Alizarin Red (Sigma) solution.

*Adipogenic differentiation*

The MSCs were seeded in T-75 flask at a concentration of 5,000 cells/cm^2^ and cultured in high-glucose DMEM supplemented with 10% FBS, 1% penicillin-streptomycin, 10 μg/ml insulin, 1 μM dexamethasone and 0.5 mM 3-isobutyl-1-methylxanthine (IBMX; all from Sigma-Aldrich) at 37^o^C in a humidified 5% CO_2_ atmosphere. After 14 days, the MSCs were stained using Oil Red O. Briefly, the cells were fixed with 10% formalin, washed with 60% isopropyl alcohol and stained with 2% Oil Red O reagent (Sigma-Aldtich). The staining was quantified by extracting the Oil Red O stain with 100% isopropyl alcohol. The incidence of cell differentiation was investigated and photographed by inverted microscope.

*Cytotoxicity evaluation of beta glucan on MSCs*

MSC proliferation and viability were determined by MTT (3-[4,5-Dimethyl-2 thiazoyl]-2,5-diphenyl-2 Htetrazolium bromide) assay (Roudbary et al., 2013). For this, cells were cultured in 96-well microplates at a density of 10^4^ cells/well. Twenty-four hours after the initial seeding, cells were either treated with extracted β (1,3) Glucan from the cell wall of *Candida albicans* in different concentration (2, 4, 8,10, 25, 50, 100, 200, 400, 800 and 1,000 μg/ml) and incubated for 48 hrs. (MSCs were culture as triplicate wells with the final volume of 100 μl/well). Then, all of the medium was removed and 10μl of MTT solution (0.5mg/ml,Sigma) and 100 μl fresh medium without FBS added to each well. After incubation for 4h at 37°C, 100 μl DMSO (Sigma) was added to each well. After incubation for 15 minutes at room temperature under smooth agitation, the plate was read immediately and the absorbance of each well was measured by ELISA reader at wavelength of 570 nm. The average values for the blank was subtracted from the average values from triplicate readings of treated wells. The experiments were repeated three times and the results of which are presented as means.

*Treatment of MSCs with optimal concentration of β (1,3) Glucan and supernatant collection*

Third subculture of MSCs were seeded at 6× 10^4^ cells per well into 6-well cell culture plates and after 24 hours treated with optimal beta-glucan concentration. For this, cell culture medium was removed and 200 μl of extracted beta-glucan and 600μl of complete culture medium was added to each well for 48 hours. Afterwards, supernatant medium was aspirated and centrifuged at 300 g for 10 minutes to remove cellular debris and stored at -80°C for fur-ther assays.

*Apoptosis assay*

Apoptosis kit (FITC Annexin V Apoptosis Detection Kit I, BD Biosciences, and USA) was used to detect apoptotic cells. The manual of the kit was strictly followed. Briefly, A549 cells were seeded in the 6-well plates (25×10^4^ cells per well). After incubation for 24h at 37°C, A549 cells were either treated (in a ratio of 1:1) with collected MSC treated supernatant from former stage. After 2 days, A549 cells were collected and washed twice with cold PBS, trypsinzed and pooled together. 5×10^5^ cells/mL of A549 cells was transferred to a tube and re-suspended in 100 μl of binding buffer. Then, 5 μl of FITC-conjugated Annexin V (Annexin V-FITC) and 5 μl of propidium iodide (PI) were added, followed by incubation (15 min at room temperature in the dark room) and at last directly analyzed by flow cytometer (Calibur, Becton Dickinson). Therefore, the pro apoptotic cells were identified as Annexin V-FITC+ and PI−. The nonviable apoptotic cells were identified as Annexin V-FITC+ and PI+ , viable cells as Annexin V-FITC− and PI− and non-apoptotic died cell as Annexin V-FITC- and PI+.

*Statistical analysis*

The experimental data were analyzed using SPSS statistical software (version 21.0) (SPSS Inc., Chicago, IL, USA) and presented as the mean ± SD from at least three independent experiments. Differences between groups were analyzed by an ANOVA; means of groups were compared by a t-test. P < 0.05 was considered statistically significant.

## Results

*Extraction and characterization of β-glucans*

**Figure1:**
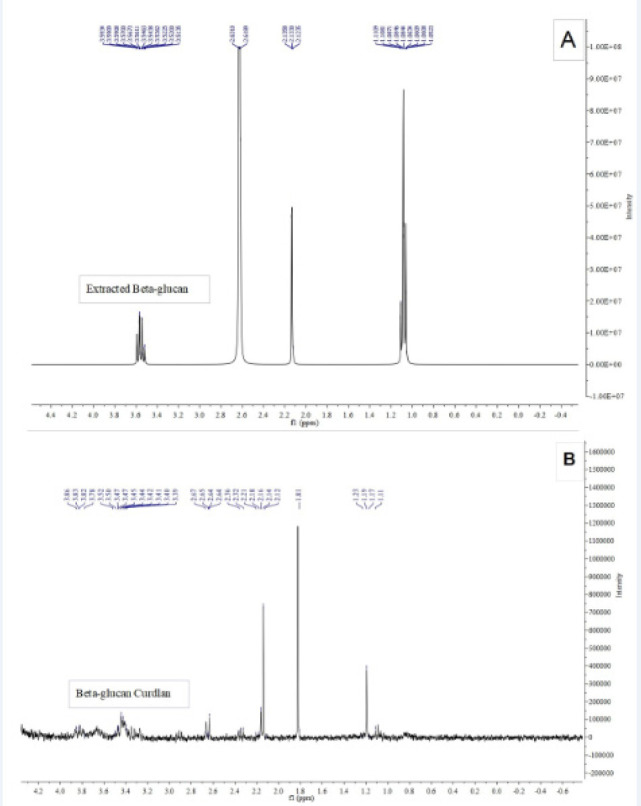
H NMR Spectrum of the Beta-Glucan Fraction of *C. albicans*

Particulate β-glucan was extracted from *C.albicans* cell wall using a combination of acid and alkaline solution. Then, the structure of β-glucan was identified with NMR. The results are shown in Figure 1.

*MSCs morphology*

**Figure 2:**
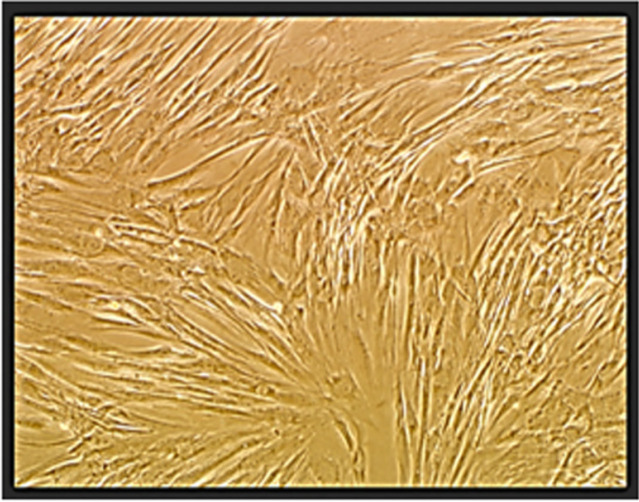
The Morphological Feature of the MSC Cells in DMEM Medium (40x magnification, passage-3)

The cultures were observed daily by phase contrast invert microscopy to examine adherent cell morphology. In the early days, individual adherent cells appeared in about 30% of the flasks and 70% of the flasks having no adherent cells. During the second week, the number of floating cell decreased within the culture medium. By the end of week 3, these cells had been completely grown and the cells were expanded by several subcultures in 25 cm^2 ^flasks, and the passage-3 were used for future examination (Figure 2). 

*Characterization of MSCs*

**Figure 3:**
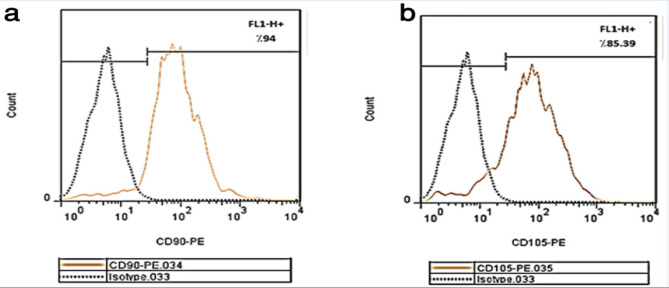
MSCs Surface Markers Expression were Analyzed by Flow Cytometry. "a shows CD90 , b shows CD105, c shows CD45 and d shows CD11-b" surface markers respectively

The MSCs were successfully and rapidly expanded into colonies of confluent spindle-shaped cells. The results of flow cytometry for CD45, CD11b , CD105 and CD90 revealed that the cells were negative for CD11b (2.13%), CD45 (1.44%) and positive for CD105 (85.39%), CD90 (94.00%). The cultured cells were considered MSCs (Figure 3).

*Analysis of adipogenic and Osteogenic differentiation*

**Figure 4:**
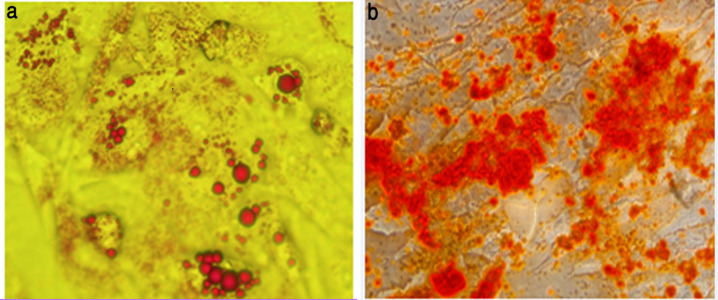
A, Adipogenic Differentiation; B, Osteogenic Differentiation (100x magnification).

Lipid vesicle accumulation in adipocytes differentiated from MSC was shown using oil red staining and the MSC adipogenic differentiation medium. The differentiated cells exhibit extensive intracellular lipid red vacuole formation typical of mature adipocytes. Alizarin Red S staining of extracellular calcium deposits was shown in mineralized MSC\derived mature osteoblasts (Figure 4).

*Effect of β-glucan on the MSCs viability*

**Figure 5.**
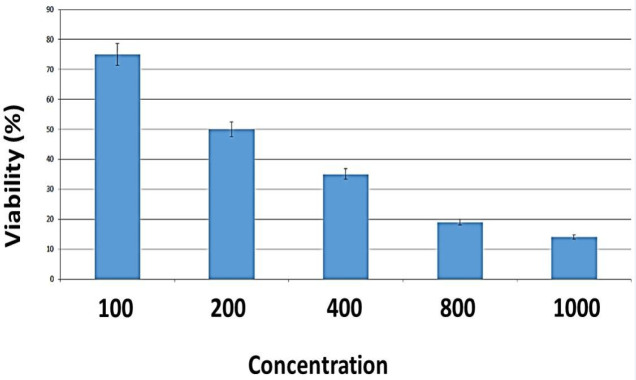
Viability Results of MSCs Treated with Different Concentration of β-glucan by MTT Test

We tested a range of β-glucan concentrations to determine the optimal concentration for MSCs treatment. MTT assay showed that exposure of MSCs to 400, 800 and 1,000 μg/ml of beta-glucan induced a significant decrease in cell viability (P<0.001). No significant changes in cell viability were observed at up to 25 μg/ml of beta-glucan (P>0.05). In addition, we found that β-glucan had a dose-dependent effect on MSCs proliferation. IC_50_ (concentration resulting in a 50% inhibition of cell growth) determine for different concentration of β-glucan. According findings IC_50_ for 1,000 μg/ml was 14%, for 800μg/ml was 19%, for 400μg/mlwas 35%, and for 200 μg/ml of β-glucan was 50%. The results showed 200 μg/ml concentration of beta-glucan determined as IC50 whereas other examined concentration were toxic for MSC and led to cell death at 400, 800 and 1,000 μg/ml after 48 hours. Finally, 200 μg/ml of beta glucan was selected for MSC treatment and apoptosis assay in this study (Figure 5).

*Cell apoptosis*

**Figure 6:**
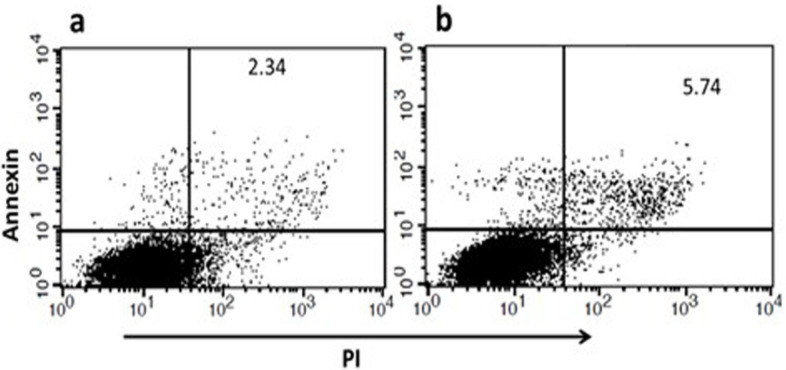
Flow Cytometry Results of the Annexin V-FITC and PI Staining for Apoptosis assay. The results of flow cytometry analysis showed that test group with MSCs supernatant treatment showed a significant increase of cell apoptosis (a) in comparison with the control group (b). P-value = 0.00093)

To investigate how treated MSC supernatant (stored culture medium of MCSs treated with β (1,3) Glucan) effect on apoptosis of A549 cells in vitro, FITC Annexin V Apoptosis Detection Kit was employed to detect apoptotic cells. The result indicated that the presence of β-glucan in the supernatant of treated test groups induced a significant increase in A549 cancer cells apoptosis compared to the control group (without beta-glucan, (P-value= 0.00093)(Figure 6).

## Discussion

Several previous studies have shown that β-Glucans can serve as biological response modifiers and induce anti-tumor properties in vitro and in vivo (Geller et al., 2019; Gao et al.,2005; Choromanska et al., 2018). Remarkably, in recent years, for anti-cancer characteristic, β-Glucans are great of consideration worldwide, however, further clinical trials and experimental investigations are still needed (Petravić-Tominac et al., 2010; Mantovani et al., 2008). On the other hand, Mesenchymal stem cells are multi-potent progenitor adult stem cells exhibit numerous beneficial properties in treatment of various diseases and therapeutic potentials due to their immunomodulatory properties (Wang et al., 2018). Despite using conventional chemotherapy regimens and radiotherapy against cancer cells; because of emerging drug resistant stemness cells, relapses and metastasis, lung cancer treatment have been faced with many obstacles in many developed and developing countries of the world. To overcome these challenges in the treatment of lung cancer, use of natural compounds and supplements like beta-glucan has received much attention for their beneficial properties (Rankin et al., 2018).

Until now, a few data are available concerning the effect of beta-glucan on lung cancer and MSC. For this reason, in the current study, we exploit the effect of β-glucans on MSCs and subsequently the possible anti-cancer property of β-glucans treated MSC supernatant was examined to suppress of lung cancer cells growth in vitro for the first time. We tested a series of β-glucans concentrations to find out the optimal concentration of β-glucans. We found that 200 μg/ml of β-glucans is the optimal concentration and was used in further tests for MSC treatment. which was able to decrease of viability of MSC significantly. surprisingly, Larguech indicated that laminarin, a beta-(1 → 3)-D-glucan, suppressed MSC proliferation and their chondrogenic differentiation which propose potential applications in clinical MSC therapy (Larguech et al., 2018). The findings strongly supposed that apoptosis of lung cancer cells was increased significantly in cells exposure to conditioned media of MSC treated with beta glucan in comparison to cells without treatments.

In agreement with our findings, Li et al., (2011) assessed the effect of hMSCs on lung cancer cell line A549 using a co-culture systems with mesenchymal stem cell supernatant (hMSCs-conditioned). They showed that hMSCs can inhibited tumor cells proliferation as compared to controls and increase the apoptosis of tumor cells. 

Similarity the beta glucan anti tumor activity, Interleukin-24(IL24) has been suggested as an effective anti-cancer agent. Zhang et al., (2013) evaluated the effects of MSCs-delivered IL24 on A549 cell proliferation and apoptosis. They observed that IL24-transduced UC-MSCs (IL24-MSCs) inhibited the growth of A549 lung cancer cells by induction of apoptosis and cell cycle arrest.

On the other hand, Murphy in another study expressed that the anti-bacterial effect of MSC is enhanced by the immunomostimulatory effect of beta-glucan and prevents acute lung injury due to infection (Murphy et al., 2017). 

The mechanisms of immune-modulatory and anti-tumor effects of beta-glucan have been explained in many studies, with focus on the major anti-tumor effects of beta-glucan being due to the immune stimulating effects of beta-glucan. One of the important inhibitory cells in the tumor microenvironment is MSCs that produce inhibitory mediators and suppress immune responses (Wang et al., 2018). In this study, the conditioned medium of MSC treated with beta-glucan modulates MSCs and enhances the anti-cancer and apoptotic effect of MSC on lung cancer cells. These effects may be due to the change in the immunological phenotype of MSCs from immunosuppressive to immune-stimulatory, and this issue can be studied in other studies.

In conclusion, our findings suggest that, conditioned media of mesenchymal stem cells treated with β-glucans is capable of inhibiting lung cancer cell lines growth. Certainly a large number of studies are urgent necessity to evaluate the β-glucans effects and function for development of novel therapeutic approaches against cancer cells in the future. 
